# A Case of Oxalate Nephropathy in a Known Diabetic Patient following Acute Alcoholic Pancreatitis

**DOI:** 10.1155/2022/6284693

**Published:** 2022-07-19

**Authors:** John Odhiambo, Hanika Patel, Anderson Mutuiri, Fazal Yakub, Ahmed Sokwala

**Affiliations:** ^1^Internal Medicine, The Aga Khan University Hospital, Nairobi, Kenya; ^2^Radiology, The Aga Khan University Hospital, Nairobi, Kenya; ^3^The Aga Khan University Hospital, Nairobi, Kenya

## Abstract

This was a case of a 39-year-old gentleman known to have diabetes mellitus since February 2021 on insulin glargine (Lantus) 16 units nocte and sitagliptin/metformin 50/500 mg once a day who presented to a tertiary teaching hospital in Kenya in May 2021 with a three-week history of vomiting and diarrhea. He had been previously admitted to a different facility with acute alcoholic pancreatitis. His examination was nonremarkable except for mild dehydration and pallor. He had moderate metabolic acidosis and deranged renal function. Prior to this, his creatinine was normal. As part of the evaluation for the rapid deterioration of renal function, a kidney biopsy performed revealed oxalate nephropathy. He was started on renal replacement therapy with hemodialysis.

## 1. Background

Oxalate nephropathy is a rare condition associated with excess oxalate in the system. This can be either from increased absorption from the gastrointestinal tract or endogenously from the liver because of glyoxylate metabolism. These are referred to as secondary and primary hyperoxaluria, respectively. Primary hyperoxaluria ensues in the background of reduced or absent activity of enzymes involved in the glyoxylate metabolism [1, 2]. In secondary hyperoxaluria, there is either increased exposure to dietary sources of oxalate or any pathological conditions that increase intestinal absorption of oxalic acid. These conditions are associated with fat malabsorption. Excess oxalate results in hyperoxaluria since it is primarily excreted by the kidneys [3]. This causes renal dysfunction, which is diagnosed primarily on kidney biopsy [4].

## 2. Case Presentation

This is a case of a 39-year-old male known to have diabetes mellitus since February 2021 on Lantus 16 units nocte and sitagliptin/metformin 50/500 mg once daily. He presented to the hospital in May 2021 with a three-week history of postprandial, nonbilious vomiting and a four-day history of watery, nonbloody diarrhea, associated with reduced appetite, progressive generalized body malaise, and reduced urine output. He had also noted decreasing insulin requirements with the last injection 2 weeks prior to presentation. The past medical history was remarkable for an admission 3 months prior to presentation and managed for acute alcoholic pancreatitis. At presentation, he was moderately pale and dehydrated, with a PR at – 87 bpm and raised BP at 157/90 mmHg. Systemic examination was nonremarkable. The initial laboratory parameters are highlighted in Tables 1 and 2.

An impression of acute kidney injury likely due to rapidly progressive glomerulonephritis with high anion gap metabolic acidosis, symptomatic uremia, and microcytic hypochromic anemia in the diabetic patient was made.

### 2.1. Initial Management

He was admitted to a high dependency unit where he underwent urgent hemodialysis, transfusion with two units of packed red blood cells, intravenous fluid resuscitation, and insulin therapy.

He also underwent lower and upper gastrointestinal endoscopy as evaluation for the anemia, which revealed gastric erosions and hemorrhoids.  Table 3 shows the results of the additional tests carried out.

### 2.2. Imaging

**Figure 1:**
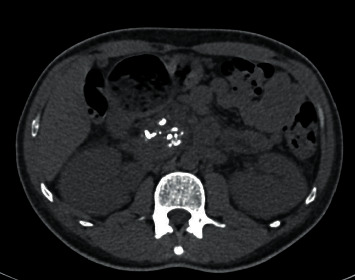
Axial noncontrast CT through the head of the pancreas shows coarse calcifications within the pancreatic parenchyma more pronounced in the head and uncinate process and are also seen in bilateral perinephric fat stranding.

**Figure 2:**
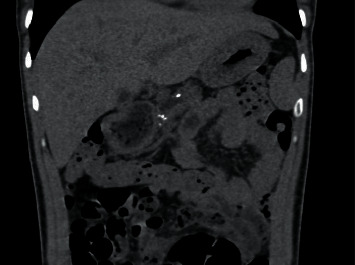
Coronal noncontrast CT through the upper abdomen shows calcifications along the body of the pancreas and mild dilatation of the pancreatic duct measuring up to 9.7 mm.

Kidney ureter and bladder ultrasound showed bilateral renal parenchymal disease. Contrast-enhanced computed tomography revealed features consistent with chronic pancreatitis; moderately edematous kidneys with moderate periphrenic stranding. The impression was revised to nondiabetic kidney disease with possibility of oxalosis because of chronic pancreatitis seen on the contrast-enhanced CT abdomen. Figures 1 and 2 show the images of the CT scans.

### 2.3. Histopathology

**Figure 3:**
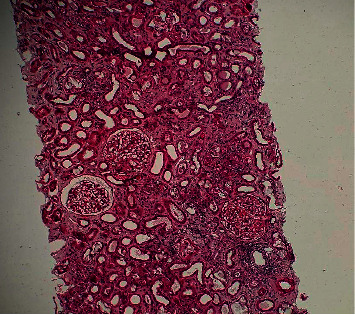
H&E (X200): diffuse acute tubular injury characterized by tubular epithelial simplification and dilation with numerous pale refractile intratubular casts.

**Figure 4:**
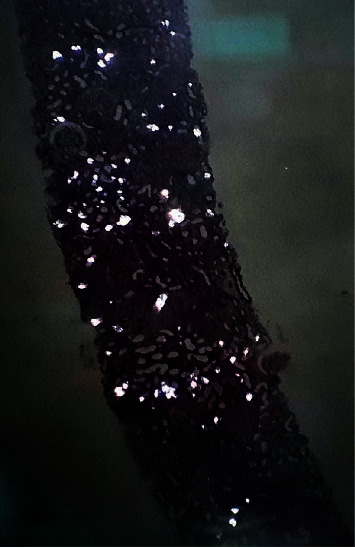
Partially polarized light microscopy (X100) shows diffuse birefringent intratubular casts.

Kidney biopsy was performed in view of the above results. This showed 1 core with 28 glomeruli, one of which was globally sclerosed. There were no segmental scars. The mesangium showed normal cellularity with a marked increase in the matrix and focal nodule formation. There was no endocapillary hypercellularity. The capillary walls showed mild thickening without spikes or double contours on silver stain. The tubules showed diffuse acute on chronic tubular injury and numerous oxalate casts on polarized light microscopy. There were severe (50%) interstitial fibrosis and tubular atrophy with chronic inflammation present in fibrotic areas. There were moderate hyaline arteriolosclerosis and arteriosclerosis. Immunofluorescence microscopy, referred out, was reported as follows: 8 glomeruli negative for immunoglobulin and complement deposits. Electron microscopy was not performed. The diagnosis was severe acute on chronic tubular injury with calcium oxalate deposits and nodular diabetic glomerulosclerosis (see Figures 3 and 4).

## 3. Discussion and Conclusion

At presentation, the aforementioned patient's renal dysfunction would have been attributed to diabetes that had been diagnosed three months before presentation. However, everything else pointed towards a possibility of nondiabetic kidney disease. He had no proteinuria, no hematuria, or any other microvascular complications and had a rapid decline of renal function, therefore, meeting the criteria for renal biopsy [5]. These, together with a previous history of acute pancreatitis and abdominal CT scan findings, raised a possibility of secondary hyperoxaluria with oxalate nephropathy. This diagnosis was confirmed on histology.

Oxalate nephropathy results from excess oxalate in the system. This can be because of increased absorption from the gastrointestinal tract, ascorbic acid administration (intravenously or orally), or increased endogenous production from defects in the enzymes involved in the glyoxylate metabolism.

Normally, oxalate present in the gastrointestinal tract is bound to calcium preventing its absorption and increasing its excretion. However, impaired exocrine function, in acute or chronic pancreatitis, leads to reduced digestion of fatty acids, hence resulting in fat malabsorption. The free fatty acids bind calcium instead, leaving the oxalate unbound and increasing its absorption into the bloodstream from the colon [4]. The excess oxalate is excreted via the kidneys causing renal damage. Mechanisms of renal damage include recurrent nephrolithiasis from calcium oxalate complexes and crystals, nephrocalcinosis, and interstitial fibrosis [6].

A systemic review on secondary nephropathy showed that 35% of the patients present with acute kidney injury followed by 29% presenting with acute on chronic kidney disease [7]. The aforementioned patient presented with acute kidney injury.

In conclusion, one needs to have a high index of suspicion for possibility of nondiabetic kidney disease in diabetic patients with rapidly deteriorating renal function, especially in the absence of other microvascular complications of diabetes.

## Figures and Tables

**Table 1:** Laboratory parameters.

Initial laboratory work-up		
Laboratory test	Results	Reference range
White blood cells (total)	6.59 × 10^9^/L	4–10
Hemoglobin	5.8 g/dl	13–18
Platelet count	241 × 10^9^/L	150–400
Creatinine	1206 *μ*mol/L	62–115
Potassium	5.78 mmol/L	3.5–5.5
Urea	36.7 mmol/L	3.2–8.2

**Table 2:** Blood gas results.

Arterial Blood Gas		
	Results	Reference range
pH	7.18	7.35–7.45
Bicarbonate	10 mmol/L	22–29
Potassium	5.7 mmol/L	3.5–4.5

**Table 3:** Additional laboratory workup.

Test	Results	Reference range
Urine albumin creatinine ratio	3.37	<3.5 mg/mmol
Transferrin saturation	14.7%	15–45%
Parathyroid hormone level	101 pg/ml	15–65 pg/ml
Vitamin	11	30–100 ng/ml
Complement 3 (C3)	Normal	
Complement 4 (C4)	Normal	
Antinuclear antibody	Negative	
Lipid profile	Normal	
Urinalysis	No hematuria/proteinuria	

## References

[1] Weigert A., Martin-Higueras C., Hoppe B. (2018). Novel therapeutic approaches in primary hyperoxaluria. *Expert Opinion on Emerging Drugs*.

[2] Belostotsky R., Frishberg Y. (2021). Novel therapeutic approaches for the primary hyperoxalurias. *Pediatric Nephrology*.

[3] Garrelfs S. F., Rumsby G., Peters-Sengers H. (2019). Patients with primary hyperoxaluria type 2 have significant morbidity and require careful follow-up. *Kidney International*.

[4] Makkapati S., D’Agati V. D., Balsam L. (2018). “Green smoothie cleanse” causing acute oxalate nephropathy. *American Journal of Kidney Diseases*.

[5] Oliva-Damaso N., Mora-Gutiérrez J. M., Bomback A. S. (2021). Glomerular diseases in diabetic patients: implications for diagnosis and management. *Journal of Clinical Medicine*.

[6] Hoppe B., Beck B. B., Milliner D. S. (2009). The primary hyperoxalurias. *Kidney International*.

[7] Lumlertgul N., Siribamrungwong M., Jaber B. L., Susantitaphong P. (2018). Secondary oxalate nephropathy: a systematic review. *Kidney International Reports*.

